# Application of Kirschner wire placement guided technology in paediatric supracondylar humerus fractures

**DOI:** 10.1186/s12891-023-07160-9

**Published:** 2024-01-12

**Authors:** Huan Liu, Lingzhi Li, Qirui Ding, Yunru GE, Ying Ding, Shouguo Wang, Haodong Fei

**Affiliations:** 1 Department of Orthopedics, The Affiliated Huaian No. 1 People’s Hospital of Nanjing Medical University, Huaian, 223300 China; 2 Department of Orthopedics, The First Affiliated Hospital of Nanjing Medical University, Nanjing, 210029 China; 3 Huaiyin Normal University, Huaian, 223300 China

**Keywords:** Kirschner wire placement guided technology, Paediatric, Supracondylar humerus fractures

## Abstract

**Background:**

To analyze the clinical efficacy of K-wire placement guided technology in paediatric supracondylar humerus fractures.

**Methods:**

A retrospective study was conducted in 105 patients who underwent closed reduction and percutaneous pinning surgeries in our hospital from June 2019 to August 2022. 54 patients treated with a assisted reduction fixation device to assist in closed reduction and percutaneous K-wire cross-fixation were allocated into the Non-guided group, and 51 patients with K-wire placement guided technology to guide K-wire placement were assigned into the Guided group. The operation duration, number of disposable K-wire placement, intraoperative fluoroscopy frequency, Baumann angle, carrying angle, fracture healing time and Flynn score of elbow joint function at the final follow-up were compared between two groups. The postoperative complications of two groups were recorded.

**Results:**

There were significant differences between two groups in terms of operation duration, intraoperative fluoroscopy frequency, and disposable K-wire placement rate (*p* < 0. 05), while no significant differences of Baumann angle, carrying angle and the fracture healing time between two groups were observed (*p* > 0. 05). In the control group, ulnar nerve injury in 2 case, pin site infection in 4 cases, mild cubitus varus in 2 cases and loss of reduction in 4 cases were detected. In the study group, ulnar nerve injury in 1 case, pin site infection in 2 cases and loss of reduction in 1 case was observed. There was no significant difference in Flynn scores between two groups.

**Conclusion:**

K-wire placement guided technology is simple and convenient. The application of K-wire placement guided technology could relatively improved disposable K-wire placement rate, shorten the intraoperative fluoroscopy frequencies and reduce complication rates**.**

## Introduction

Supracondylar humerus fractures (SHFs) are the most common elbow injury in children, accounting for approximately 15% of all childhood fractures [1]. According to Gartland staging, SHFs can be split into three categories [2], with extensional SHFs accounting for more than 90% of cases in children [3]. Closed reduction and percutaneous pinning(CRPP) is the classic surgical procedure for the treatment of SHF in children [2], which is a stable fixing technique, according to biomechanical studies, however there is still a risk of postoperative fracture re-displacement [4]. An earlier study found that up to 18% of postoperative fracture re-displacements with CRPP occurred [4]. One probable explanation for this is that intraoperative pin repositioning, which is always necessary even under fluoroscopic guidance, can cause a loss of the pull-out resistance of almost 50% [5]. Simultaneous multiple pin repositioning will increases radiation exposure as well as the potential of neurovascular injury [6, 7]. We developed the K-wire targeting device (Patent No. ZL 202220185369. 4) for use in pediatric CRPP surgery and assessed its clinical efficacy in order to decrease intraoperative pin repositioning.

## Materials and methods

### Inclusion and exclusion criteria

Inclusion criteria: (1) age ≤ 14 years; (2) fresh Gartland type II and type III SHF (time from injury to operation < 5 days); (3) surgical method to CRPP; (4) follow-up ≥ 6 months.

Exclusion criteria: (1) open SHF; (2) inability to perform normal elbow exercises; (3) preoperative severe neurovascular injury; (4) preoperative or postoperative diagnosis of a disease affecting bone development or fracture healing, such as vitamin D deficiency, severe malnutrition, malignant tumor, hypothyroidism, etc.; (5) inability to complete a full follow-up.

### Ethics approval statement

Our study was approved by the Ethics Committee of The Affiliated Huaian No. 1 People’s Hospital of Nanjing Medical University(Number: KY- 2023–130-01). All guardians of the children were informed about the purpose of the study and the study procedure and provided their informed consent.

### General information

Retrospective analysis was performed on the clinical data of children with extensional SHF treated in our institution from June 2019 to August 2022. This study comprised 105 patients in total, with 62 males and 43 females, ages 1 to 13 (6. 055 ± 3. 215), that met the aforementioned criteria. The study group and the control group of patients were separated and Table 1 displays the general facts about the two groups. In terms of gender, age, fracture type, side of injury, time from injury to surgery, and cause of injury, there were no statistically significant differences between the two groups (*p* > 0. 05).

**Table 1:** Comparison of general data of extensional SHF between two groups of children

Group	Gender		Age, year (mean ± SD)	Side		Time^a^, hour (mean ± SD)	Gartland type	
	Male	Female		Left	Right		Type II	Type III
Guided, *N* = 51	28	23	6.24 ± 3.172	20	31	48.02 ± 29.504	29	22
Non-guided, *N* = 54	34	20	5.87 ± 3.257	24	30	52.56 ± 23.141	35	19
t/χ^2^	0.705		0.850	0.295		-0.873	0.697	
*P* value	0.401		0.562	0.587		0.385	0.404	

^a^from injury to operation

### Surgical methods

**Fig. 1:**
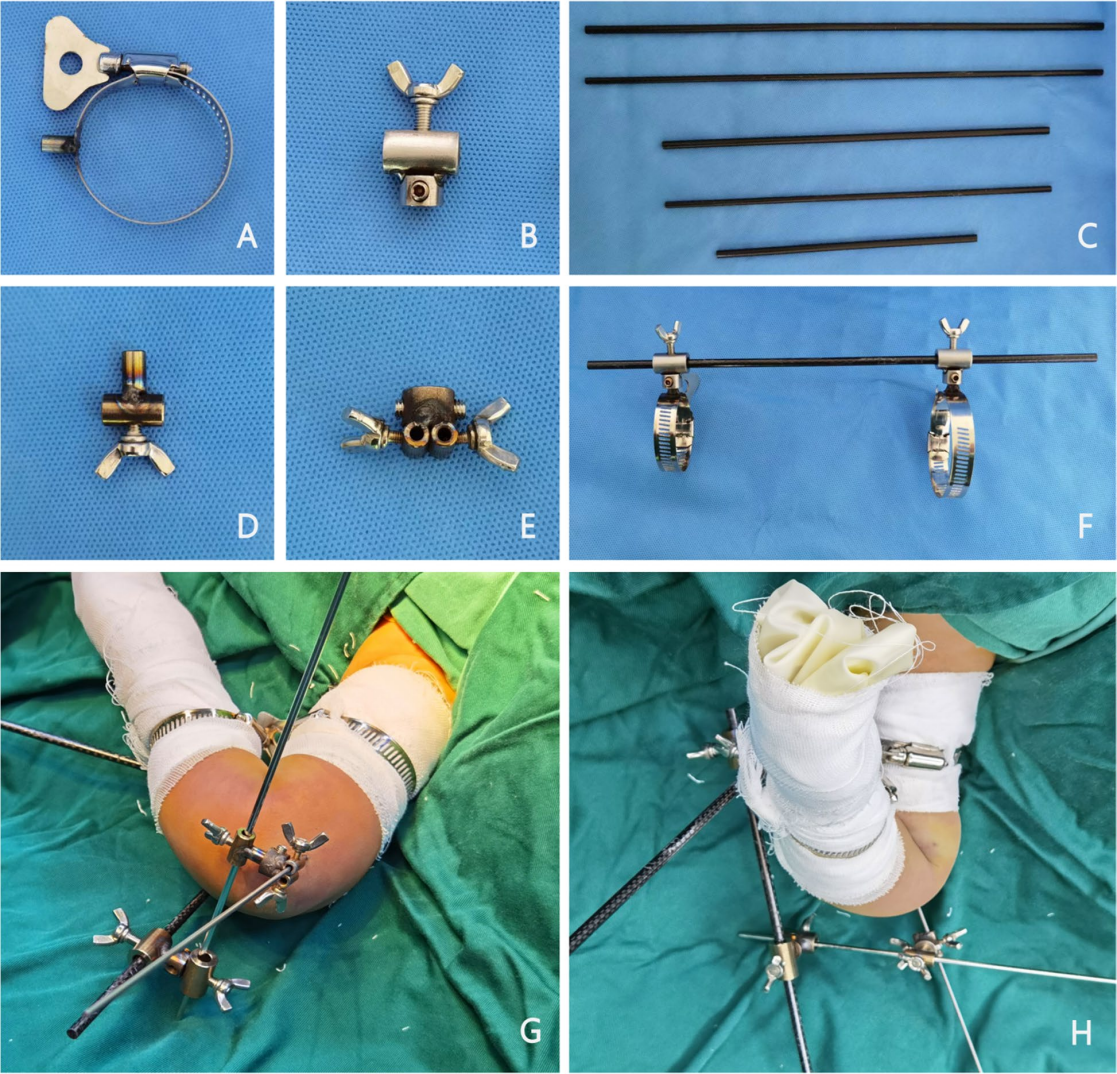
Usage of K-wire aiming device, connection mode, and auxiliary reduction and fixation device during operation. **A **A metal handle can be used to adjust a clamp with a 6 mm diameter side convex column clamp that comes in three sizes: 21-44 mm, 27-51 mm, and 46-70 mm; **B** A self-made universal fixed concave parts that have an inner diameter of 6 mm and the clamp with a side convex column may be added to it. The top butterfly buckle can be used to tighten a carbon fibre rod or a K-wire; **C** Different length carbon fibre rods in the 5 mm or 6 mm range; **D** Self-made universal fixed convex with a convex column diameter of 6 mm that can connect to another self-made aiming component or another universal fixed concave. The bottom butterfly buckle can tighten a carbon fibre rod or a K-wire; **E** Self-made aiming equipment with a concave diameter of 6 mm, a self-made universal fixed convex, an aiming sleeve inner diameter of 2. 5 mm, and a side butterfly buckle that can tighten and fix the K-wire. **F** With the universal fastening concave connection shown in the schematic picture of the device connection, the clamp can be rotated and fixed 360 degrees; **G**, **H** A schematic showing how to operate the K-wire aiming device

All CRPP operations were performed by the same physician team. The K-wire placement guided technique for paediatric supracondylar humerus fracture reduction and fixation has been authorized for patenting under utility model innovation with patent number ZL202220185369. 4 (Fig. 1).

#### Non-guided group:

During general anesthesia, children's lens, thyroid, and gonads are shielded by lead clothes. The forearm and upper arm are secured by two stainless steel clamps, and traction is used to realign the fracture. With the aid of a steering fixation device, the two stainless steel clamps are attached to the carbon fibre rod for temporary fixation to maintain reduction of fractured end. The fracture reduction was confirmed with fluoroscopy of elbow joint anteroposterior and lateral, and if necessary, fluoroscopic medial and lateral oblique films were used to monitor the medial and lateral column reduction. If there is a considerable rotational or translations displacement, repeat the fracture reduction methods described above. The anterior–posterior displacement of the fracture can be modified by adjusting the length of the steering fixing device in respect to the carbon fiber connecting rod if the fluoroscopy only shows a slight anterior–posterior displacement. To modify the modest rotational displacement of the medial and lateral columns, one can rotate the forearm anteriorly or posteriorly by loosening the stainless steel clamp on the forearm. Two K-wires are inserted under fluoroscopic guidance at the medial and lateral condyles of the humerus once the fracture has been sufficiently reduced. The results of the fluoroscopic examination are utilised to estimate the extent of the pin entry. Determine the entrance location using fluoroscopy after first inserting the K-wire into the skin and making contact with the bone surface. The K-wire is inserted into bone by the electric drill at a slow speed until a sence of "breakthrough" is felt. Check the K-wire position through fluoroscopy and reposition the pin if necessary. Bend the K-wire and snip off the end of the pin after making sure the elbow joint is firmly fastened. After the surgery, immobilize the elbow joint using a polymer splint.

#### Guided group:

**Fig. 2:**
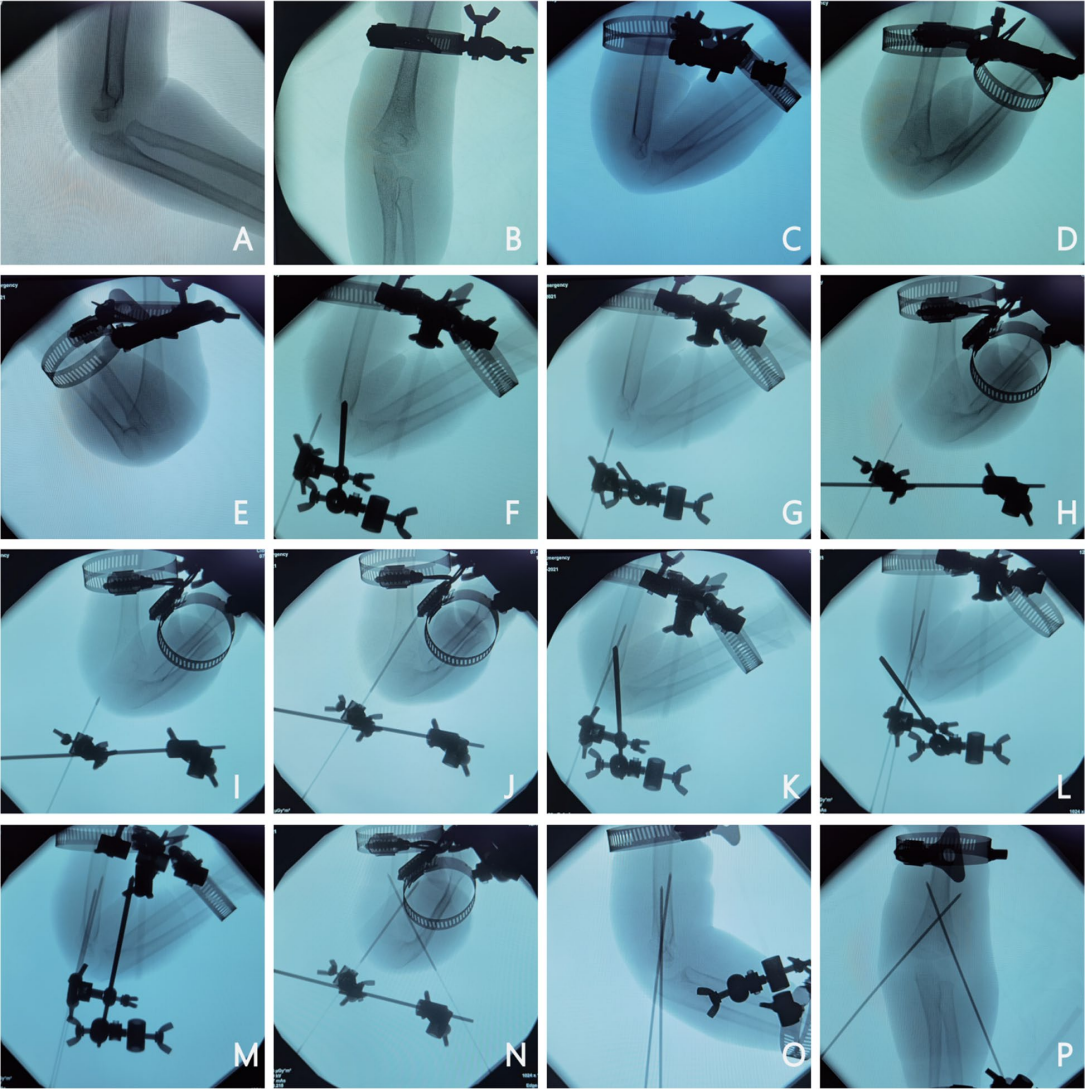
Perspective view of the use of auxiliary reset fixture and K-wire aiming device in guided group. **A**, **B** Prior to surgery, Gartland II SHF was visible in the results of anterior and lateral fluoroscopy; **C**, **D** The elbow joint was rotated while the manual reduction device was temporarily fixed. The fracture's broken end was well-reduced, as evidenced by the fluoroscopy's anterior and posterior positions as well as medial and lateral oblique positions; (F, G) Adjusting the K-wire's entry point and angle in the sagittal plane in vitro: Lateral film view; **H**, **I** Adjusting the K-wire's entry point and angle in the coronal plane in vitro:Inside oblique view; **J**, **K** The medial condylar K-wire is in the previous aiming direction, as shown in the lateral view and medial oblique film; **L**-**N** lateral K-wire was inserted in the same way; (O, P) Fluoroscopy is used to determine whether the fixation is secure once the temporary fixation has been removed

Preoperative attachment of the K-wire placement guided device and the auxiliary reduction and fixation device. As in the non-guided group, the fracture was realigned and temporarily immobilized. The K-wire should be fixed in the aiming sleeve, the aiming device adjusted, the pin placed outside the skin near the point of entry, the elbow joint fluoroscopically visualized in the anteroposterior and lateral position, and the point and direction of entry ultimately determined based on the fluoroscopy results. Finally, the electric drill should be connected, and the K-wires should be cross-inserted at a low speed guided by the direction of the aiming sleeve until a "breakthrough" is felt (Fig. 2).

The polymer splint was immobilised in both groups for 3 to 4 weeks post-operatively. The timing of removing the bilateral K-wire and polymer splint was determined by the weekly reviews of post-operative x-rays. After the splint was taken off, the functional flexion and extension activities were begun.

### Evaluation indicator

Operative time, the number of K-wires put at once, the number of intraoperative fluoroscopies, Baumann's angle, carrying angle, and fracture healing time were all noted as perioperative data. Postoperative complications included ulnar nerve injury, cubitus varus, elbow valgus deformity, pin site infection, and number of re-displacements after fixation. The Flynn elbow function score [8], as detailed in Table 2, was utilised at the final follow-up to assess the overall excellent rate.

**Table 2:** Flynn elbow function evaluation criteria

Rating	Cosmetic Factor: Carrying-Angle Loss	Functional Factor: Motion Loss^a^
Excellent	0° ~ 5°	0° ~ 5°
Good	5° ~ 10°	5° ~ 10°
Fair	10° ~ 15°	10° ~ 15°
Poor	> 15°	10° ~ 15°

^a^limitation of flexion and extension of elbow joint

### Statistical analysis

SPSS 21. 0 software was used for data analysis. The measurement data were expressed by (x̅ ± s). When the data were normally distributed, one-way ANOVA was used for inter-group comparison, LSD method was used for pairwise comparison; and paired T-test or one-way ANOVA was used for intra-group comparison. When the data are not normally distributed, the rank sum test was used. Chi-square test or Fisher accurate test was used for counting data. Rank sum test was used to compare the rank data. *P* < 0. 05 was considered a statistically significant difference.

## Results

**Fig. 3:**
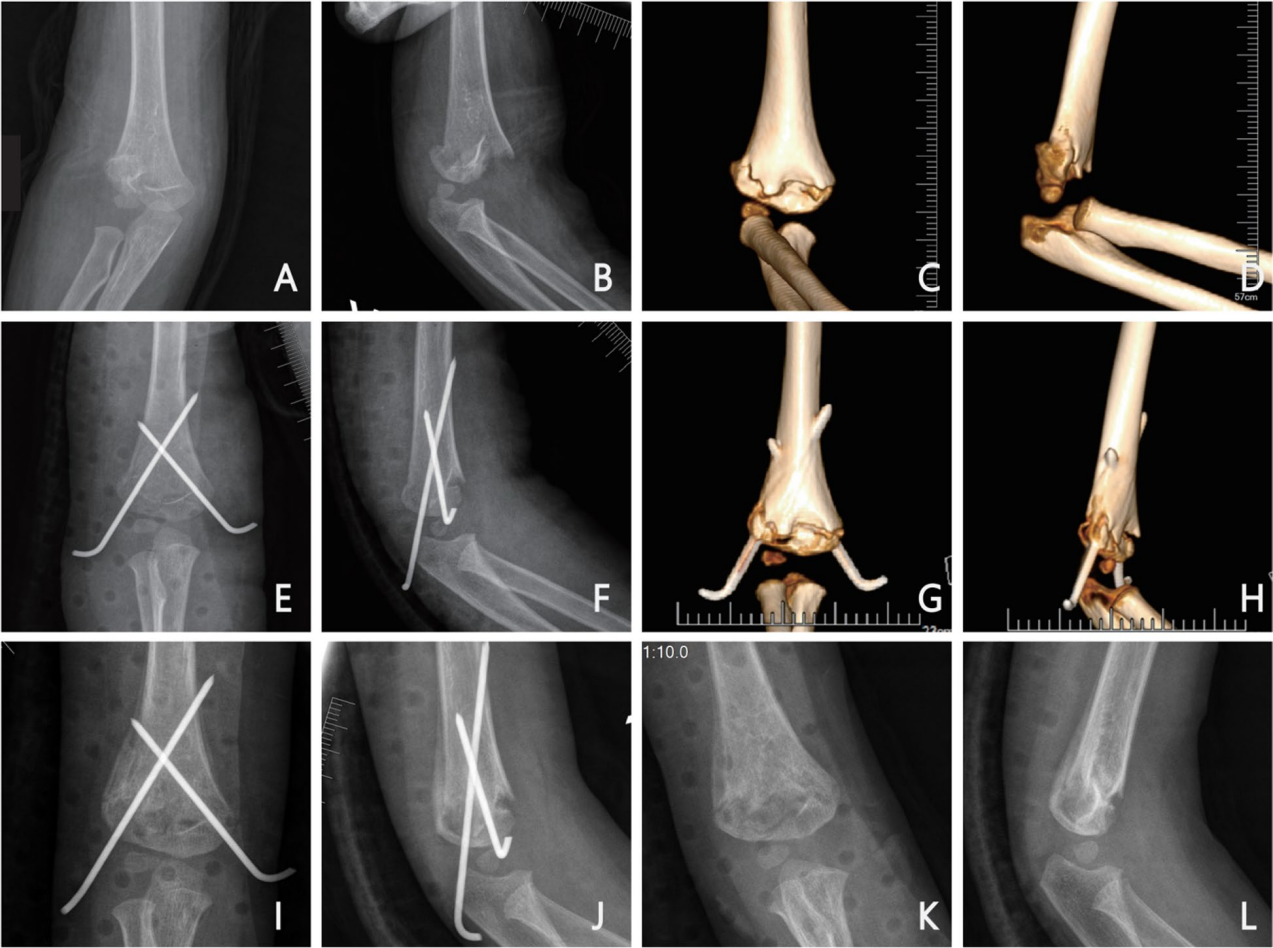
A Typical case of guided group. The patient had right-sided Gartland type II SHF and was a 2-year-old boy. **A-D** Preoperative positive X-ray and three-dimensional CT images that are positive Gartland type II SHF. **E–H** One day following surgery, the fracture had been successfully reduced and fixed with K-wire; **I-L** The fracture line had blurred three weeks after the operation, and the K-wire had been taken out

Both patient groups successfully underwent the procedure. As detailed in Table 3, while there was no statistical difference in Baumann angle or fracture healing time between the two groups (*p* > 0. 05), there were statistically significant differences in the operation time, intraoperative fluoroscopy times, and carrying angle of the study group compared to the Non-guided group. (A typical case is shown in Fig. 3).

**Table 3:** Comparison of perioperative data and fracture healing time between two groups

Group	Operation time^a^, min^c^	Fluoroscopy times, time^c^	Rate^b^	Baumann angle^c^	Carrying angle^c^	Fracture healing time, week^c^
Guided, *N* = 51	31.27 ± 4.920	15.53 ± 2.063	83/19	72.31° ± 1.794°	10.00° ± 1.483°	3.96 ± 0.848
Non-guided, *N* = 54	38.72 ± 4.249	20.69 ± 2.126	21/87	72.56° ± 1.777°	10.17° ± 1.463°	4.13 ± 0.778
t/χ^2^	-8.316	-12.600	80.478	0.828	0.731	0.403
*P* value	< 0.001	< 0.001	< 0.001	0.489	0.564	0.290

^a^time from the auxiliary reduction device installation to the K-wire tail cutting

^b^for needle placement one / more times

^c^mean ± SD

### Follow-up results

All patients in both groups were followed up for 8-17 months (11. 67 ± 3. 192) months. As shown in Table 4, there was no significant difference in the excellent and good rate between the two groups in Flynn score(*P* > 0. 05).

**Table 4:** Comparison of Flynn scores between the two groups

Group	Excellent	Good	Fair	Poor	Rate^a^
Guided, *N* = 51	33	14	3	1	92.16%
Non-guided, *N* = 54	30	17	5	2	87.04%
χ^2^	0.733				
*P* value	0.392				

^a^for total excellent and good rate

### Complications

In the non-guided group, there were two cases of ulnar nerve injury, with neurological symptoms going away three days after removal of the K-wire; four cases of pin site infection, which resolved after removal of the K-wire; two cases of mild cubitus varus, which worked well and weren't treated; and four cases of postoperative fracture displacement, of which two required additional CRPP treatment. In the guided group, there were one case of ulnar nerve damage, two cases of pin site infection that cleared up after removal, and one case of postoperative fracture displacement. In neither group were there any elbow valgus abnormalities. The incidence of complications in the guided group was significantly lower than that in the non-guided group (χ^2^ = 3. 873, *p* < 0. 05).

## Discussion

SHF is the most common fracture of the elbow joint in children. For the treatment of SHF, Gartland I can be managed with plaster fixation alone; Gartland II and III are often managed with CRPP, and open reduction and fixation are required if closed reduction fails. Moreover, it has been claimed that closed reduction and plaster fixation for Gartland II can produce outcomes comparable to those of CRPP, however there is a chance of re-displacement and cubitus varus following surgery [9, 10]. A unilateral placement technique and a cross-placement approach are the two categories for K-wire placement in CRPP. Both methods can achieve sufficient biomechanical stability, and there is no difference between them [11]. For the fracture to remain mechanically stable and to minimize trauma, the K-wire must be placed precisely. Some researchers have used a hypodermic injection pin to guide the point and direction of K-wire insertion on unstable SHFs, which can significantly reduce the need for K-wire resets. However the technique requires the K-wire to be placed at a certain depth under the skin, which increases surgical trauma, and only works on the lateral side of the elbow, with a risk of nerve injury when applied medially [10]. In this study, the CRPP is fitted with the self-designed and manufactured device that can help to assist in the reduction and temporarily fixate the SHF during the surgery. In addition to assisting in pin pointing the entrance site and orientation, the aiming device can also be used to realize the precise positioning of the K-wire. The study's findings revealed that the guided group's rate of K-wire placement at once was 81. 37% much higher than the non-guided group's rate of 19. 44%. The use of the device lessens harm to the soft tissue and bone near the pin site in addition to significantly reducing intraoperative reinsertion.

Children and the surgeon will both be exposed to more radiation from SHFs with large displacement and difficult repositioning [12]. According to a prior research, between 30. 7 and 126 fluoroscopies were performed during the CRPP [13, 14]. In addition, when the C-arm image intensifier was used as the operating table, the radiation exposure of the elbow and neck of the patient was significantly higher [6, 12]. In multi-directional unstable SHFs, we should apply a joystick technique to assist in the reduction of the broken end of the fracture [15], which can significantly increase the likelihood that the reduction will be successful, increase the effectiveness of the procedure, and simultaneously decrease the need for fluoroscopy. Rotating the child's arm for lateral imaging usually results in loss of repositioning of these fractures due to high instability [16]. Our technology reduces needless, repetitive manual reduction procedures by providing a robust temporary fixation and fluoroscopic insertion of the elbow joint without displacement the broken end. The K-wire can be placed precisely thanks to the targeting device, which significantly lowers the likelihood that it will reset. The results of the study indicate that the number of surgical fluoroscopies was about 18, which is less than previously reported. In addition, the operator's exposure to radiation is significantly reduced by fluoroscopy because it does not require constant movement and allows for operator concealment behind a lead screen.

To evaluate the reduction of the broken end of the fracture, the standard anteroposterior and true lateral views of the elbow joint are typically sufficient. The anterior humeral line, which in a normal elbow joint should travel through the middle third of the ossification center of the lateral humeral condyle, is the primary anatomical landmark evaluated on lateral films [16]. It happens frequently that the severity of a fracture is underestimated due to subpar radiographic methodology. Consequently, careful assessment of the repositioning should be performed prior to the placement of the K-wire. We recommend a fluoroscopic medial and lateral tilt view prior to K-wire placement to assess the repositioning of the medial and lateral columns of the distal humerus and to ensure that rotation is corrected. To get a better placement position and angle, a 3D model reconstruction of the distal humerus is required to identify the placement point and the range of placement angles [7]. We routinely perform preoperative 3D CT and design the K-wire placement point and angle according to the direction of the fracture line in order to obtain more stable fixation. Nonetheless, fluoroscopic intraoperative guidance and the operator's skill both play a role in the ultimate K-wire placement. At the same time, the fixation of high one-time pin placement rate increases the anti-pullout ability of K-wire [5]. According to the study's findings, the guided group had a reduced likelihood of postoperative re-displacement than the non-guided group. However, for a novice, it might be challenging to spatially comprehend the swollen child's elbow and its intricate anatomy. Placing the K-wire can also be challenging, particularly if the relocation is unstable and the surface markers are difficult to reach. The difficulty of putting the K-wire during surgery is significantly reduced by the use of repositioning fixation and aiming devices for the K-wire placement, which can be mastered by novice and experienced surgeons with simple training.

Ulnar nerve damage is more likely to occur with cross pinning [17–19]. To prevent ulnar nerve damage, which surely increases surgical trauma, a minor medial incision has been utilized to expose the medial pinning point [20]. In order to protect the ulnar nerve, we locate it by palpation when soft group swelling is not immediately apparent and avoid the ulnar nerve row area when inserting the pin. However, this is not a completely safe technique [21]. Before inserting the K-wire with a low speed drill, we prefer to first puncture the skin with the tip of the K-wire and then execute blunt row stripping around the entry point with the tail of the wire. Previous studies have shown that among 1541 patients in supine position, 69 (4. 5%) suffered from a ulnar nerve injury [22], while our results show a lower ulnar nerve damage, 3 cases of 105(2. 9%).

## Conclusion

To summarize, the K-wire placement guided technique is straightforward and simple to learn, and it is deserving of clinical application in children with supracondylar humerus fractures to increase the rate of pin placement at once, decrease the number of intraoperative fluoroscopy, and lower the incidence of complications.

Some limitations of this study should be mentioned. Firstly, this study is its retrospective design, which is more susceptible to bias than prospective study designs. Another limitation may be the short follow-up period. The average follow-up time in our study was 6 months, although this is comparable to other publications involving SCHF results. Finally, the sample size is too small. A Prospective, large-sample studies with long-term follow-up will be conducted in the future to further demonstrate its effects and values.

## Data Availability

The datasets used or analyzed during the current study are available from the corresponding author on reasonable request.
